# Special Issue: “Smart and Functional Polymers”

**DOI:** 10.3390/molecules24162976

**Published:** 2019-08-16

**Authors:** Xiangru Feng, Mingqiang Li, Yang Li, Jianxun Ding

**Affiliations:** 1 Key Laboratory of Polymer Ecomaterials, Changchun Institute of Applied Chemistry, Chinese Academy of Sciences, Changchun 130022, China; 2 Department of Biomedical Engineering, Columbia University, 116th and Broadway, New York, NY 10027, USA; 3 Laboratory for Biomaterials and Drug Delivery, Boston Children’s Hospital and Harvard Medical School, 300 Longwood Ave., Boston, MA 02115, USA

**Keywords:** polymerization or post-polymerization modification methods, polymer-based supramolecular chemistry, stimuli-responsive polymers, shape memory polymers, self-healing polymers, polymers for industrial catalysis, polymers for water or effluent treatment, polymers for sensing, separation, and purification, polymers for fabrication, renewable polymer materials used for agriculture, functional polymers used in food science, polymers for information storage, electronics, and energy conversion, functional polymers for diagnosis, imaging, drug delivery, and tissue engineering, polymers with biological activity (e.g., antitumor, antidiabetic, and antimicrobial activity), polymer-based medical devices

Polymerization provides an efficient strategy for synthesizing macromolecules with versatile functionality [1,2]. Smart and functional polymers with various active groups have attracted increasing interest as they hold considerable promise for a variety of applications [3,4]. The advanced polymers can be constructed via the polymerization of functional monomers or post-polymerization modification [5,6,7]. These polymers possess a combination of the physical properties of nanoscale or microscale architectures and the physiochemical reactivities of the attached functional groups [8]. Moreover, their ability to form microscopic and macroscopic assemblies in response to external targets or signals gives them unique physiochemical properties (e.g., a large surface-to-volume ratio, variable composition and size, dynamic association, and reversible phase separation) and tailored functionalities (e.g., enhanced sensitivity and specificity, extraordinary target binding affinity, and tunable surface chemistry), which are absent in small molecules [9,10].

Eleven original research articles and six review papers have been collected in this Special Issue. These articles focus on functional polymers with specific structures and performances.

Six original research articles focus on the synthesis and characterization of advanced functional polymers. Chen et al. synthesized an amphiphilic polyurea consisting of cyclohexyl-*tert*-butyl polyurea and poly(ethylene glycol) (PEG) for the encapsulation of chemotherapeutic drug paclitaxel (PTX) [11]. The PEGylated polyurea micelle showed more efficient delivery of PTX into 4T1 cells, with enhanced antitumor efficacy. Guerrero et al. proposed a method to purify poly(vinyl alcohol)−polyaniline (PVA−PANI) copolymers at different aniline concentrations [12]. After activation with glutaraldehyde exposure, reduction of the polymer was detected, along with an increase of the benzenoid section of PANI. Ndugire et al. developed carbohydrate-grafted glycopolymers via a catalyst-free perfluorophenyl azide-mediated Staudinger reaction [13]. Using this method, they successfully conjugated maltoheptaose and mannose onto poly(lactic acid). Valero and co-workers prepared different polyurethanes with castor oil and isophorone diisocyanate by adding polycaprolactone diol and chitosan [14]. The change of polyols from using castor oil significantly increased the mechanical properties of interest. Guo et al. immobilized three polyether imidazole ionic liquids onto ZSM-5 zeolite to acquire three immobilized catalysts [15]. The prepared catalysts maintain excellent stability and high catalytic activity after eight cycles. Wen, Chen, and co-workers reported the purification and characterization of a water-soluble exopolysaccharide (EPS-2) from the fermentation culture of endophytic fungus CSL-27 of saffron [16]. It was found that EPS-2 could protect cochlear hair cells from ototoxicity exposure.

Five original research articles report polymers with specific structures and performances, including core−shell structure and stimuli-responsive properties. Nanoparticles with a core−shell structure have shown advantageous performance. Xu et al. used cheap rare earth hydroxide as a precursor to develop a monodisperse hexagonal NaYF_4_:Yb^3+^/Ln^3+^ core and NaYF_4_:Yb^3+^/Ln^3+^@NaGdF_4_ core−shell nanoparticles with well-controlled shapes [17]. The sizes of the nanocrystals were tunable, and the core−shell nanoparticles showed intense emission under 980-nm laser excitation. Kredel et al. prepared core−shell particles with highly fluorinated shell materials [18]. The incorporation of fluoropolymers into core−shell particle structures can be used for the melt/shear organization technique, which produced free-standing fluoropolymer opal and inverse films with different hydrophobic properties and reflection colors. Tang et al. used bovine serum albumin to fabricate vitamin E (VE)-albumin core−shell nanoparticles for the delivery of PTX and VE [19]. Owing to the existence of VE as an oil core, the nanoparticles achieved higher PTX-loading efficiency and also overcame the P-gp-mediated drug efflux. Stimuli-responsive (e.g., pH, temperature, and light) polymers have shown great promise in the design of smart materials for various biomedical applications, such as drug delivery and molecular imaging. Fan and co-workers developed reversible pH-responsive copolymers by using tertiary amine-based monomers, 2-(dibutyl amino)ethyl methacrylate, and 2-(dimethylamino)ethyl methacrylate [20]. These polymers were pH-sensitive and could be responsively fine-tuned in aqueous solution. At low pH, the polymers were in unimer state, while a high pH would lead to polymer aggregation. The p*K*_a_ values of these polymers fall into the physiological pH range, making them ideal carriers for therapeutic drugs and imaging contrast agents to the tumor microenvironment or cytosol. Han and co-workers synthesized a series of methoxy poly(ethylene glycol)−poly(L-alanine) thermosensitive hydrogels with different degrees of polymerization (DPs) [21]. They found that hydrogels with higher DPs had better gelation ability than those with lower ones. 

In addition, six review articles summarize the current improvements in advanced polymer-based materials for various applications. Lu et al. introduced anhydrous electrorheological materials, fabricated from conducting polymers and nanocomposites [22]. They mainly focused on the study of the electrical conductivity and thermal or mechanical stability of nanocomposites. Due to the emergence of various reversible materials bearing reversible-covalent linkages, reversible sol−gel transition, or reversible bonds, there has been a rapid development in reversible polymerization in recent years. In this context, Tang et al. summarized recent progress made in this area and provided insight into future reversible polymerization systems [23]. In order to investigate the effect of drug ratios used in polymer nanoparticles, Pan et al. reviewed polymer-based co-delivery systems and drug combinations for synergistic antitumor efficacy [24]. They pointed out that understanding the drug ratio of therapeutic agents and heterogeneity of tumors helped with optimizing the therapeutic effect. In another review, Tang et al. highlighted recent advances in pH-responsive nanomaterials in cancer diagnosis and treatment [25]. They summarized the recent advances in polymer design, mechanistic investigation, drug delivery, and bioimaging applications. Wang et al. reviewed the recent development of phenylboronic acid-based glucose-sensitive gels for self-regulated drug delivery, which might promote a drug delivery system for diabetes therapy [26]. In another review article, Narayanaswamy and Torchilin discussed methods of manipulating hydrogels for targeted drug delivery in diverse diseases [27].

Smart and functional polymer materials represent an interdisciplinary field that integrates physics, chemistry, material science, engineering, and biology. Over the past decade, the field has experienced rapid progress as a result of unmet needs in various areas. This Special Issue aims to provide a comprehensive collection of the latest advances in the development of synthetic approaches, the mechanisms underlying structure-property correlations, and the current and emerging applications of smart and functional polymers. The issue covers smart and functional polymers for a diverse range of applications, involving synthetic chemistry, materials science, and biomedical technology. It mentions state-of-the-art breakthroughs that will provide guidance and references for interested readers.
